# HIV prevalence in persons with severe mental illness in Uganda: a cross-sectional hospital-based study

**DOI:** 10.1186/1752-4458-7-20

**Published:** 2013-07-17

**Authors:** Patric Lundberg, Noeline Nakasujja, Seggane Musisi, Anna Ekéus Thorson, Elizabeth Cantor-Graae, Peter Allebeck

**Affiliations:** 1 Division of Social Medicine, Department of Public Health Sciences, Karolinska Institute, Widerströmska Huset, Tomtebodavägen 18A 8th floor, 171 77 Stockholm, Sweden; 2 Department of Psychiatry, College of Health Sciences, Makerere University, Kampala, Uganda; 3 Division of Global Health/IHCAR, Department of Public Health Sciences, Karolinska Institute, Stockholm, Sweden; 4 Division of Social Medicine and Global Health, Department of Clinical Sciences, Lund University, Malmö, Sweden

**Keywords:** Prevalence, HIV, Mental illness, Psychosis, Low-income country, Uganda

## Abstract

**Background:**

In Uganda, a previous study reported high HIV prevalence in persons with severe mental illness (SMI) compared to the general population, suggesting that persons with SMI might constitute a high-risk group for HIV. However, the study included first-time psychiatric admissions only, a group whose HIV prevalence may not reflect the prevalence in persons with SMI in general. We determined prevalence and correlates of HIV in both first-time and previous psychiatric admissions, in a psychiatric hospital in Uganda.

**Methods:**

Cross-sectional study of HIV status in persons consecutively discharged from psychiatric admission wards in Butabika hospital, Uganda. Inclusion criteria: age 18–49 years; schizophrenia, bipolar disorder, depression, or other non-substance-use-related psychosis; Luganda or English proficiency. Exclusion criterion: Mental incapacity to give informed consent. Participants were HIV-tested, and interviewed using a structured questionnaire. Data were analysed using logistic regression.

**Results:**

HIV prevalence was 11.3% (CI 8.8-13.8) overall, 7.3% (CI 4.1-10.5) in men and 14.3% (CI 10.6-18.0) in women. Females had higher risk of HIV infection than males (OR 2.10; CI 1.20-3.67), after adjustment for age. Older patients had higher risk of HIV infection than younger patients (40–49 vs. 18–29 years: OR 2.34; CI 1.27-4.32), after adjustment for sex. Place of residence, marital status, income, education, occupation, psychiatric diagnosis and history of previous admission were not associated with HIV infection, after adjustment for sex and age. The above associations did not significantly differ between men and women.

**Conclusions:**

Persons admitted for SMI in Uganda have higher HIV prevalence than persons in the general population, irrespective of previous admissions. The excess HIV prevalence is mainly confined to women. The findings call for the integration of HIV prevention, testing and care with mental health services in settings with generalized HIV epidemics. Moreover, further research is needed to clarify the mechanisms underlying the increased HIV prevalence in women with SMI in Uganda, and to identify effective community-based interventions for this vulnerable group.

## Introduction

Studies of HIV prevalence in persons with severe mental illness (SMI) in sub-Saharan Africa provide a diverse picture. Some studies have reported HIV prevalence estimates below or similar to those in the general population [1-3], while others have reported estimates higher than those in background populations [4,5]. The reason for the variation in HIV prevalence across studies is unknown, but overall, might be due to sampling variability, context-specific selection processes (since studies were hospital-based), and differential survival of persons with SMI who are HIV infected. In addition, the observed variation between studies might indeed reflect true variation in the incidence of HIV in persons with SMI between contexts.

In Uganda, Maling et al. [5] reported that HIV prevalence in persons with SMI was more than two times that in the general population at the time of the study (18.4% vs. 8.5%). Intrigued by these findings, we performed a qualitative study in persons with SMI, documenting stories of casual sex during mental illness episodes, non-monogamous sexual partners, exploitation by sexual partners, rape by non-partners, and involuntary sexual inactivity [6]. In the light of Maling et al.’s study, our qualitative findings from Uganda raise the question as to whether persons with SMI might be a group at particularly high risk of HIV in Uganda. It should be noted, however, that Maling et al.’s study [5] differs from other sub-Saharan African studies in one important aspect; the study targeted first-time psychiatric admissions only. Thus, as suggested by the authors, reverse causality between SMI and HIV (SMI secondary to HIV) might have inflated HIV prevalence estimates, given that cases of SMI secondary to HIV may be over-represented in samples of first-time psychiatric admissions [7,8]. In contrast, persons previously admitted may on average have had a longer duration of mental illness, and therefore have a greater probability of mental illness onset preceding any potential HIV infection. Thus, the HIV prevalence in this group may potentially provide a more representative picture of HIV prevalence in persons living with SMI in general.

In this study, we assessed HIV prevalence in both first-time and previous psychiatric admissions, in a psychiatric hospital in Uganda. In addition, informed by our previous qualitative findings [6], we investigated whether demographic characteristics, socio-economic position, and psychiatric characteristics were associated with HIV infection, and further, whether these associations differed between men and women.

## Methods

### Study design

Cross-sectional study of prevalence and correlates of HIV in persons consecutively discharged from male and female psychiatric admission wards in Butabika hospital, Uganda.

### Setting

Butabika hospital is situated in the outskirts of Kampala. It is a national referral facility with a catchment area including all of Uganda. Kampala residents are over-represented in the hospital, due to access barriers for persons residing in rural Uganda, e.g. transportation costs.

Patients are generally brought to Butabika hospital by relatives or by the police. Psychiatric patients are admitted to male and female psychiatric admission wards, or to male and female forensic psychiatric admission wards (as general psychiatric patients), depending on the day of the week. The hospital also comprises convalescent wards and a somatic ward for physically ill psychiatric inpatients. The hospital has approximately 500 beds for adults. Treatment and discharge decisions are taken by psychiatrists, or by psychiatric clinical officers (PCOs).

According to Ugandan national policy, HIV counselling and testing should be routinely offered to patients attending health services [9]. At the time of the study, this policy was not yet fully implemented in Butabika hospital.

### Participants

We aimed at a minimum sample size of 500 persons with 250 men and 250 women, in order to be able to estimate an HIV prevalence of 12% ± 3%, and to be able to detect a difference in HIV prevalence of 8% between two equally sized sub-groups.

Inclusion criteria were: (1) age 18–49 years, (2) diagnosis of schizophrenia, bipolar affective disorder, depression, and other non-substance-use-related psychosis, (3) knowledge of English or Luganda (the main language in central Uganda). The age range was chosen in order to target adults in reproductive age.

Exclusion criterion was: Mental incapacity to give informed consent. Mental capacity was assessed twice, first by the discharging clinician, and second, by the research assistant. Mental capacity was defined as: Capacity to understand the information about the study, capacity to express a choice based on this information, insight about having a mental health problem, and capacity to participate in a focussed discussion.

The inclusion of participants started on February 8th 2010 and continued for both men and women in parallel until the sample size had been accrued in both groups, i.e. on April 4th 2010. All consecutive patients leaving the two psychiatric admission wards during this period were screened for eligibility. Patients leaving the wards had one of three possible destinations: home, convalescent ward, or somatic ward. Patients leaving to home were approached for potential participation prior to their departure. Patients transferred to convalescent or somatic wards were followed-up and approached when cleared for discharge from their new ward.

### Data collection

We trained five nurses and one PCO from Butabika hospital to work as research assistants. First, a one-day training session in seminar form was held. Second, pilot interviews with subsequent feed-back were conducted. Third, a one week full-scale pilot trial was performed, with observation and evaluation of study procedures.

A structured questionnaire covering participants’ demographic, socio-economic and psychiatric characteristics was used. The questionnaire was translated into Luganda and independently back-translated, modified and pilot tested.

Interviewing and blood sampling were performed in one session, in secluded rooms inside the hospital premises. Pre-test counselling was given by research assistants as part of the informed consent procedure. Structured questions were read aloud by research assistants. Research assistants performed blood sampling and delivered samples to the hospital laboratory. Test results disclosure and post-test counselling were performed the same day, or when participants preferred, at a later occasion.

The first and second authors supervised the data collection and held weekly meetings with research assistants for debriefing and guidance, and for collection of questionnaires and test results. Incomplete questionnaires were returned to participants for completion, when possible.

### Measures

#### HIV status

An HIV testing algorithm widely used in Uganda was employed: HIV screening was performed using the rapid test Determine HIV-1/2 (Abbott Laboratories, Illinois, USA). If the test result was negative the participant was diagnosed HIV negative. If positive, a second rapid test was performed, HIV 1/2 Stat-Pak Dipstick (Chembio Diagnostic Systems Inc., New York, USA). If both tests were positive the participant was diagnosed HIV infected. If results were discordant a third rapid test was performed, Uni-Gold Recombigen (Trinity Biotech, Wicklow, Ireland), and the result of this test used to determine the diagnosis. Participants having a previous positive HIV test result in their patient file were not tested again.

#### Demographic characteristics

Four demographic variables known to be associated with HIV status in the general population [10] were used: sex, age, urbanicity of place of residence (urban = Kampala, semi-urban = townships/municipalities, rural = villages), and marital status.

#### Socio-economic position

Three indicators of socio-economic position were used [11]. Education was operationalized as highest attained education, not counting repeat years in school. Income was operationalized as regularity of cash income prior to admission, and was dichotomised: no income vs. irregular/regular income, given few persons reporting regular income. Occupation was operationalized as occupation prior to admission, and was dichotomised: no employment vs. any employment.

#### Psychiatric characteristics

Diagnoses made by psychiatrists or PCOs at discharge were extracted from clinical charts. Diagnoses were not strictly coded according to DSM-IV or ICD-10, and were therefore categorised into three broad categories: bipolar affective disorder, non-affective psychosis, and depression. Thus, diagnoses containing the words ‘affective’, ‘bipolar’, ‘mania’, were categorised as bipolar affective disorder. Of the remaining diagnoses, those containing the word ‘depression’ were categorised as depression. The remaining diagnoses were categorised as non-affective psychosis. Psychiatric history was operationalized as previous admission for mental illness: yes vs. no.

#### Year of SMI onset

Year of SMI onset was obtained through self-report.

#### Year of HIV diagnosis

In persons who were found HIV-infected, self-reported information about previous positive HIV-tests was used in order to obtain the year of first HIV diagnosis.

### Statistical analysis

Data was entered using EpiData (Epidata Association, Odense) and independently checked against original questionnaires. Statistical analyses were performed using SPSS 20 (SPSS Inc., Chicago). Missing data was marginal; the variable with most missing data was ‘occupation’ (n = 10, 1.7%).

HIV prevalence estimates with 95% confidence intervals were calculated as described by Rothman [12]. Associations of demographic, socio-economic, and psychiatric variables with HIV status were investigated using logistic regression. Adjustments were made for confounding by *a priori* selected variables sex and age, by simultaneously entering these variables as covariates into logistic regression models. Interaction between each variable and sex was investigated by fitting logistic regression models with and without multiplicative interaction terms, and comparing the goodness-of-fit of the models using the likelihood-ratio test.

HIV prevalence estimates for the general population were derived from the 2011 Uganda AIDS Indicator Survey (UAIS) [10]. The UAIS is a nationally representative survey of HIV prevalence in 20433 randomly selected individuals aged 15–49 years.

In order to ensure comparability, we calculated HIV prevalence estimates standardised for age, using the UAIS sample as standard population. Definitions of age strata, and weights for strata, were derived from table 8.3, page 104 [10]. Given the definitions of strata, standardization could only be performed for persons aged 20–49 years. Total HIV prevalence for persons in this age range in the UAIS was also calculated for comparison, using data from the above table.

Sensitivity analyses were performed in order to explore the potential influence of selection bias caused by inclusion of persons having SMI secondary to HIV into the study sample. Based on a previously used definition of mania secondary to HIV [7,8,13], we defined the broader concept SMI secondary to HIV as: (1) HIV-infected, (2) first admission for SMI, (3) no family history of SMI. HIV prevalence was then re-calculated in a restricted sample where persons having SMI secondary to HIV had been excluded.

### Ethical considerations

All study procedures were approved by the Makerere University Medical School Research and Ethics Committee and by the Uganda National Council of Science and Technology.

Consent forms clarified that participation was voluntary, that participation would not in any way influence the future care given, and that participants had the right to withdraw at any time. Before informed consent was sought, participants were informed that their HIV test result would be disclosed to the psychiatrist or PCO in charge. Participants diagnosed HIV-infected were referred to the HIV clinic at Butabika hospital, where antiretroviral medication was available for free throughout the study period. Participants received 5000 Ugandan shillings (2.5 US dollars) as compensation for their time.

## Results

During the study period, 626 persons (275 men, 351 women) left male and female psychiatric admission wards in Butabika hospital, and met inclusion criteria. Of these, 8 persons (1.3%; 8 men) were judged incapable of giving informed consent, and were excluded. Thus, 618 persons (267 men, 351 women) were invited for participation. 16 persons (2.6%; 8 men, 8 women) declined participation. The final sample consisted of 602 persons (259 men, 343 women).

### Prevalence of HIV

68 persons (19 men, 49 women) were diagnosed HIV-infected. HIV prevalence was 11.3% (95% CI 8.8-13.8) overall, 7.3% (95% CI 4.1-10.5) in men and 14.3% (95% CI 10.6-18.0) in women. Table 1 presents HIV prevalence according to background characteristics.

**Table 1:** **Prevalence of HIV according to background characteristics in 602 men and women with severe mental illness**, **consecutively discharged from psychiatric admission wards in Butabika hospital**, **Uganda**

	**Men**			**Women**			**Total**		
	**n**	**Cases**	**Prev**	**n**	**Cases**	**Prev**	**n**	**Cases**	**Prev**
**Demographic characteristics**									
Sex									
Male							259	19	**7.3**
Female							343	49	**14.3**
Missing							0	0	
Age									
18-29	120	5	**4.2**	152	19	**12.5**	272	24	**8.8**
30-39	85	7	**8.2**	113	13	**11.5**	198	20	**10.1**
40-49	53	7	**13.2**	76	17	**22.4**	129	24	**18.6**
Missing	1	0		2	0		3	0	
Place of residence									
Urban	54	4	**7.4**	71	8	**11.3**	125	12	**9.6**
Semi-urban	94	6	**6.4**	85	16	**18.8**	179	22	**12.3**
Rural	107	9	**8.4**	184	24	**13.0**	291	33	**11.3**
Missing	4	0		3	1		7	1	
Marital status									
Never married	139	5	**3.6**	163	19	**11.7**	302	24	**7.9**
Ever married	119	13	**10.9**	180	30	**16.7**	299	43	**14.4**
Missing	1	1		0	0		1	1	
**Socioeconomic position**									
Income									
No income	132	7	**5.3**	200	29	**4.5**	332	36	**10.8**
Any income	123	10	**8.1**	142	20	**14.1**	265	30	**11.3**
Missing	4	2		1	0		5	2	
Education									
None to primary 7	123	8	**6.5**	207	33	**15.9**	330	41	**12.4**
Secondary 1 or higher	135	11	**8.1**	135	15	**11.1**	270	26	**9.6**
Missing	1	0		1	1		2	1	
Occupation									
Unemployed	58	3	**5.2**	181	24	**13.3**	239	27	**11.3**
Any employment	201	16	**8.0**	152	21	**13.8**	353	37	**10.5**
Missing	0	0		10	4		10	4	
**Psychiatric characteristics**									
Psychiatric diagnosis									
Bipolar affective disorder	142	10	**7.0**	195	27	**13.8**	337	37	**11.0**
Non-affective psychosis	109	9	**8.3**	115	17	**14.8**	224	26	**11.6**
Depression	6	0	**0.0**	33	5	**15.2**	39	5	**12.8**
Missing	2	0		0	0		2	0	
Psychiatric history									
Previously admitted	189	14	**7.4**	217	29	**13.4**	406	43	**10.6**
Not previously admitted	70	5	**7.1**	126	20	**15.9**	196	25	**12.8**
Missing	0	0		0	0		0	0	
**TOTAL SAMPLE**	**259**	**19**	**7.3**	**343**	**49**	**14.3**	**602**	**68**	**11.3**

### Correlates of HIV

Females had higher risk of HIV infection than males, after adjustment for age (OR 2.10; 95% CI 1.20-3.67), see Table 2. Patients of higher age (40–49 vs. 18–29 years) had higher risk of HIV infection than persons of lower age, after adjustment for sex (OR 2.34; 95% CI 1.27-4.32). Urbanicity of place of residence, marital status, income, education, occupation, psychiatric diagnosis and history of previous admission were not associated with HIV status, after adjustment for sex and age.

**Table 2:** **Associations of demographic characteristics**, **socio**-**economic position and psychiatric characteristics with HIV status**, **in 602 men and women with severe mental illness consecutively discharged from psychiatric admission wards in Butabika hospital**, **Uganda**

	**Proportion HIV**-**infected (%)**	**OR (95% CI)**	**aOR**^**1**^** (95% CI)**
**Demographic characteristics**			
Sex			
Male	19/259 (7.3)	1	1
Female	49/343 (14.3)	2.11 (1.21-3.67)**	2.10 (1.20-3.67)*
Age			
18-29	24/272 (8.8)	1	1
30-39	20/198 (10.1)	1.16 (0.62-2.17)	1.15 (0.62-2.16)
40-49	24/129 (18.6)	2.36 (1.28-4.35)**	2.34 (1.27-4.32)**
Place of residence			
Urban	12/125 (9.6)	1	1
Semi-urban	22/179 (12.3)	1.32 (0.63-2.78)	1.31 (0.61-2.80)
Rural	33/291 (11.3)	1.20 (0.60-2.42)	1.06 (0.52-2.16)
Marital status			
Never married	24/302 (7.9)	1	1
Ever married	43/299 (14.4)	1.95 (1.15-3.30)*	1.60 (0.92-2.79)
**Socioeconomic position**			
Income			
No income	36/332 (10.8)	1	1
Any income	30/265 (11.3)	1.05 (0.63-1.76)	1.04 (0.62-1.77)
Education			
None to primary 7	41/330 (12.4)	1	1
Secondary 1 or higher	26/270 (9.6)	0.75 (0.45-1.26)	0.86 (0.51-1.47)
Occupation			
Unemployed	27/239 (11.3)	1	1
Any employment	37/353 (10.5)	0.92 (0.54-1.56)	1.02 (0.58-1.81)
**Psychiatric characteristics**			
Psychiatric diagnosis			
Bipolar affective disorder	37/337 (11.0)	1	1
Non-affective psychosis	26/224 (11.6)	1.07 (0.63-1.81)	1.06 (0.61-1.82)
Depression	5/39 (12.8)	1.19 (0.44-3.24)	0.99 (0.36-2.74)
Psychiatric history			
Previously admitted	43/406 (10.6)	1	1
Not previously admitted	25/196 (12.8)	1.23 (0.73-2.09)	1.28 (0.74-2.21)

Odds ratios have been obtained using logistic regression analysis.

^1^Adjusted for confounding by sex and age.

* *p* < 0.05, **p < 0.01.

Tests for interaction with sex were non-significant for all variables. Thus, logistic regression analyses stratified for sex were not performed.

### Comparison with national data on HIV prevalence

HIV prevalence in men, i.e. 7.3% (95% CI 4.1-10.5), was broadly similar to that reported for men in the general population: 6.1% (95% CI 5.5-6.7) [10]. HIV prevalence in women, i.e. 14.3% (95% CI 10.6-18.0), was higher than that reported for women in the general population 8.3% (95% CI 7.6-9.1) [10]. It should be noted however, that the age range in the current study (18–49 years) was different from that in the general population sample (15–49 years).

Table 3 presents HIV prevalence for persons with SMI and persons in the general population, in corresponding strata of age and sex (note: ages 20–49 only, due to the age categorisation used in the UAIS report). Age-standardised HIV prevalence in persons with SMI (aged 20–49 years) was 12.0% overall, 7.7% in men, and 15.5% in women. For comparison, HIV prevalence in this age range of the general population was 8.8% overall, 7.5% in men, and 9.9% in women [10].

**Table 3:** HIV prevalence in corresponding age strata in persons with severe mental illness and in persons in the general Ugandan population

	**Total**		**Men**		**Women**	
	**Current study**	**UAIS**	**Current study**	**UAIS**	**Current study**	**UAIS**
	**N** = **557**	**N** = **15108**	**N** = **241**	**N** = **6618**	**N** = **316**	**N** = **8490**
**Age group**	%	%	%	%	%	%
20-24	10.9	5.4	4.2	2.8	16.1	7.1
25-29	9.2	7.4	3.6	4.0	13.8	9.8
30-34	10.1	10.2	8.0	9.1	11.9	11.0
35-39	10.1	11.6	8.6	11.0	11.1	12.1
40-44	19.7	11.0	12.9	11.3	25.7	10.7
45-49	17.5	10.4	13.6	10.2	19.5	10.5
**Total**	**12.0**^**1**^	**8.8**	**7.7**^**1**^	**7.5**	**15.5**^**1**^	**9.9**

General population data has been derived from the 2011 Uganda AIDS Indicator Survey (UAIS). Note: Only participants aged 20–49 years are included in this table.

^1^HIV prevalence standardized for age, using the 2011 Uganda AIDS Indicator Survey as standard population.

### Temporal relationship between SMI onset and HIV diagnosis

The year of SMI onset pre-dated the year of HIV diagnosis in 41 participants (15 men, 26 women). The year of SMI onset was the same as the year of HIV diagnosis in 15 participants (3 men, 12 women). The year of SMI onset post-dated the year of HIV diagnosis in 11 participants (1 man, 10 women). For one HIV positive person (woman) information about the year of SMI onset was missing.

### Sensitivity analyses

Sensitivity analyses were performed in order to explore the potential influence of selection bias due to inclusion of persons with SMI secondary to HIV into to the sample. Persons with SMI secondary to HIV were excluded (for definition, see Methods), and HIV prevalence estimated anew. HIV prevalence in this restricted sample (n = 588) was: 9.2% (95% CI 6.9-11.5) overall, 7.0% (95% CI 3.9-10.1) in men, and 10.9% (95% CI 7.5-14.3) in women.

## Discussion

Age-standardised HIV prevalence was 12.0%, i.e. higher than the prevalence in the corresponding age range in the Ugandan general population: 8.8% [10]. The current study thus confirms previous findings of increased HIV prevalence in persons with SMI in Uganda, although the current estimate is lower than previous findings [5]. Further, the study extends previous knowledge by demonstrating an increased HIV prevalence not only in first-time psychiatric admissions, but also in persons previously admitted. This distinction is important, since persons previously admitted may have had a longer duration of mental illness, which increases the probability that their mental illness onset pre-dated any potential HIV-infection. The influence of selection bias due to reverse causality between SMI and HIV may therefore be decreased in the current study, compared to in the previous Ugandan study, which specifically targeted first-time psychiatric admissions [5].

Women had significantly higher HIV prevalence than men. Indeed, comparison of HIV prevalence between persons with SMI and persons in the general population suggested that HIV prevalence is increased in women with SMI, but not in men. An increased sex gap in HIV prevalence in persons with SMI (women > men) was noted also by Maling et al. [5]. Women in Uganda face sexual health risks due to low power in decision-making about sex [14], male partner non-monogamy [15,16], and intimate partner violence [17,18]. Our previous qualitative study [6] illustrated how SMI may exacerbate Ugandan women’s existing sexual vulnerability. Thus, a woman unable to earn own income due to psychiatric symptoms, and with poor or ambivalent support from relatives due to mental illness stigma, may not have other options than staying in a risky sexual relationship. Similarly, a woman with impaired risk perception during illness episodes may be an easy prey for potential rape perpetrators [6]. The current study suggests that women with SMI may be at increased risk of HIV in this low-income sub-Saharan African setting, although longitudinal studies are needed to confirm this notion.

Of particular note, HIV prevalence was elevated also in young women (age 18–29) with SMI. In the Ugandan general population, young women have higher HIV prevalence than young men [10] partly due to cross-generational sex [19]. The current findings warrant concern about a further increased sexual vulnerability in young women with SMI.

Overall, older persons had higher HIV prevalence than younger persons, similar to the pattern in the general population [10], although at a closer examination, this pattern was more consistent in men. HIV is a chronic condition, and the absence of a higher HIV prevalence in women aged 30–39, than in women aged 18–29 years, might raise questions about the mortality of young women with SMI who are HIV-infected, although changes in HIV incidence over time could also explain this finding. Antiretroviral treatment is necessary for long-term survival with HIV. In resource-poor settings, persons with SMI face increased obstacles to both access and adherence to such treatment. For instance, SMI contributes to poverty [20], and poverty is a barrier to treatment access in Uganda [21,22]. In addition, poor coverage of psychiatric services leaves many persons with SMI untreated, and psychiatric symptoms can interfere with ability to adhere to antiretroviral treatment [23,24]. A facility-based Ugandan study found no difference in mortality between HIV-infected persons with and without SMI [24], but the mortality of HIV-infected persons with SMI in the Ugandan population remains unknown.

Indicators of socio-economic position were not significantly associated with HIV status, similar to the previous Ugandan study [5]. However, careful inspection of descriptive results (Table 1) provides some suggestion of sex-specific associations, although tests for interaction were non-significant: HIV infection tended to be associated with high socio-economic position in men, but with low socio-economic position in women. Interestingly, previous qualitative findings illustrated how lack of income may increase motivation for sex in women (sex in exchange for money/treatment), and decrease opportunity for sex in men (work needed to attract partners) [6]. Potentially, a larger study would have been able to detect sex-specific associations between socio-economic position and HIV.

Psychiatric diagnosis was not associated with HIV status. In most previous studies from sub-Saharan Africa (but see [25]), no difference has been found in HIV prevalence between persons having different primary psychiatric disorders, i.e. schizophrenia, bipolar disorder, and depression [1-3,5], while several studies have reported high HIV prevalence in persons having ‘organic affective disorder’, ‘delirium’ [5], ‘psychotic disorder due to general medical condition’ [2] ‘and psychosis not otherwise specified’ [25], potentially due to inclusion of cases of SMI secondary to HIV into these categories. In the current study, we deemed the available chart diagnoses of HIV-associated mania/psychosis/depression unreliable, given the limited diagnostic modalities used at Butabika hospital. Therefore we decided to use broad diagnostic categories without any etiologic assumption, and chose to explore the potential influence of SMI secondary to HIV on results in sensitivity analyses. Thus, the diagnostic categories used in the current study were crude, and clinical heterogeneity within categories could clearly have contributed to the lack of association between diagnosis and HIV status. An alternative interpretation, however, is that what matters for HIV risk in Uganda is not the type of SMI one has, but rather the fact that one does have SMI. Hypothetically, the effects of psychiatric symptoms on behavior could have less impact on HIV risk than the more general consequences of having a SMI, such as downward social drift and stigma.

### Limitations

The study was hospital-based and results may not apply to persons with SMI in Uganda in general. Personal or family characteristics influencing help-seeking, access, or referral to mental health services could be associated with HIV risk, thus causing selection bias. Conversely, access to mental health services may lead to improved clinical state for persons under treatment, potentially decreasing HIV risk, leading to lower HIV prevalence in the current sample, than in persons with SMI in the community. A population-based approach would have been required in order to avoid these potential biases.

The definition of SMI secondary to HIV used in sensitivity analyses has unknown predictive value in the Ugandan context, although is based on previously used research criteria for mania secondary to HIV [7,8,13]. Consequently, results of sensitivity analyses should be interpreted with caution. In generalized HIV epidemics, such as the Ugandan HIV epidemic, many persons are HIV-infected at low age, before the typical age of onset of primary SMI. Given the current definition of SMI secondary to HIV (i.e. HIV-infected, first admission for SMI, no family history of SMI), in Uganda many persons with primary SMI may be falsely classified as having secondary SMI. Thus, the use of the current definition may overestimate the selection bias caused, leading to an overly conservative estimate of the true HIV prevalence in persons with primary SMI.

Psychiatric diagnoses were extracted from clinical charts, and were not confirmed e.g. using a structured clinical interview. Misclassification of diagnosis could potentially have contributed to the lack of association found between psychiatric diagnosis and HIV status.

A small proportion of patients invited refused to participate (2.6%; 8 men, 8 women). Hypothetically, if persons knowing, or suspecting, that they were HIV positive preferentially refused in order to avoid stigmatisation, the current HIV prevalence would be an underestimate.

## Conclusion

Persons admitted for SMI in Uganda have higher HIV prevalence than persons in the general population. HIV prevalence is high both in persons previously admitted, and in first-time psychiatric admissions. The excess HIV prevalence is mainly confined to women.

The findings suggest that mental health services in Uganda, and other countries with generalized HIV epidemics, need to take into account the frequent comorbidity of SMI with HIV. HIV risk assessment, HIV counseling and testing, and HIV treatment and care should be integrated with mental health services. Conversely, HIV treatment programmes should take into account the high burden of mental illness among persons with HIV, and integrate mental health assessments and interventions with routine HIV care.

More generally, the current findings add to previous concerns about sexual vulnerability, and high HIV risk, in women with SMI. Research is needed to clarify the mechanisms underlying the increased HIV prevalence in women with SMI in Uganda. Further, given that most women with SMI in Uganda may not be reached solely relying on the limited mental health services available, research should identify effective community-based interventions, potentially including social protection and stigma reduction components, tailored for this vulnerable population.

## Competing interest

The authors declare that they have no competing interests.

## Authors’ contributions

PL designed the study, collected data, analysed data, and wrote the manuscript. NN participated in data collection. SM participated in designing the study. AT participated in designing the study and in interpreting the results. ECG and PA participated in the design of the study, in data analysis, in interpretation of results and in manuscript writing. All authors read and approved the final manuscript.
